# Generation and Analysis of High-Gain Orbital Angular Momentum Vortex Wave Using Circular Array and Parasitic EBG with oblique incidence

**DOI:** 10.1038/s41598-017-17793-1

**Published:** 2017-12-12

**Authors:** Rui Xi, Haixia Liu, Long Li

**Affiliations:** Key Laboratory of High Speed Circuit Design and EMC of Ministry of Education, School of Electronic Engineering, Collaborative Innovation Center of Information Sensing and Understanding, Xidian University, Xi’an, 710071 China

## Abstract

This paper presents an effective method for high-gain orbital angular momentum (OAM) vortex wave generation based on the integration of a circular antenna array (CAA) with a parasitic electromagnetic band gap (EBG) structure, which is referred to as the EP-CAA. The resonant height of the EBG structure at different oblique incidences is analyzed parametrically based on the defect mode transmission mechanism to achieve reasonable predictions of a consistent 3-dB gain bandwidth with optimal gain enhancement for different OAM modes. The effective radiation aperture of the EP-CAA at oblique incidence is proposed for analytical calculation of the aperture efficiency of the OAM beams (OAM-AE). A Wilkinson power divider (W-PD) is designed to extend the operating bandwidth of the EP-CAA, and the proposed W-PD arrangement is applicable for feasible OAM modes. Fabricated prototypes of the EP-CAA carrying four OAM modes operating at 10 GHz are measured to verify the effectiveness of the theoretical analysis, the maximum realized gain for different OAM modes are confirmed to be enhanced by at least 6 dB in 5% 3-dB gain bandwidth. The divergence angles of different OAM modes can be effectively concentrated using the proposed EP-CAA.

## Introduction

Nowadays, the precious radio spectrum resource suffers from transmission congestion as the demand for communication rate even-increasing^1–3^. Electromagnetic waves carrying orbital angular momentum (OAM) can be utilized to provide a major increase in the number of transmission channels available for the communications^4–12^ with a theoretically unlimited number of orthogonal OAM modes. Therefore, OAM has recently become a frontier research subject among scholars worldwide owing to its promising applications in extending the communication channel capacity and improving the spectral efficiency^13^. Moreover, it was also demonstrated recently that OAM can not only be applied in the optical frequency regime^7–12,14^, but also be used in the low frequency radio domain^15,16^. Since then, many OAM generation techniques have been introduced for further development of OAM-carrying radio beams, with techniques that utilize metasurfaces to transform plane or spherical waves into OAM waves^17–21^_,_ elements of the circular antenna arrays (CAAs) fed with uniform amplitude but with successive phase differences^22–25^, and fiber-based approaches with helical gratings to generate and exchange the OAM states^20,21^. OAM beams are characterized by radiating beams that have helical phase fronts with the azimuthal phase term exp(*ilφ*) (where *l* is the topological charge, and *φ* is the azimuthal angle around the propagation axis). OAM beams of higher orders show larger divergence. As a consequence, an efficient approach to focus the divergent beam and reduce the null region becomes an urgent issue for OAM beams to maintain their rotating wave front performance over a long transmission distance. There have already been several attempts to focus the divergent OAM beams based on the concentration effect of parabola, including those of spiral reflectors^26^ and spiral parabolic antennas^27^. However, it is not only difficult to generate OAM waves with different values of topological charge *l* due to the complex operating principle of these devices, but it is also difficult to manufacture the required devices because of their specifically curved structures. Recently, W. L. Wei *et al*. proposed a novel high gain vortex wave generation based on Fabry-Perot cavity theory^28^, in which a highly-reflective surface served as the superstrate, which represents pioneering research on the subject of high gain vortex wave generation. However, the published work, which described only a single high gain OAM mode operating at a single frequency, did not provide a systematic analysis or design guidelines for gain enhancement of different OAM modes with different oblique main lobe directions. To date, there have been no systematic reports on predictable design guidelines for the gain enhancement and the 3-dB gain bandwidths of different OAM modes with different oblique main lobe directions.

In this paper, characteristics of the electromagnetic band gap (EBG) structure with different oblique incidence angles are analysed systematically using the theory of defect mode transmission^29^ to provide a reasonable and convincing design prediction of the high gain achievement and 3-dB gain bandwidth for arbitrary OAM modes. A novel and detailed design process to achieve high gain performance for arbitrary OAM modes characterized by their varied main lobe angles is also provided. The inter-slab separation, the resonant height and the number of unprinted slabs of the EBG structure with oblique incidences are parametrically analyzed to achieve the optimal high gain achievement. Then, high gain verification simulation is performed for seven OAM modes (*l = *−3, −2, −1, 0, 1, 2, 3) based on the integration of the well-designed parasitic EBG superstrate and a microstrip patch CAA, the structure is termed the EP-CAA. The aperture efficiency of the OAM beams (OAM-AE) is defined analytically based on the proposed effective radiation aperture of the EP-CAA with different incident angles in this paper. The gain enhancement is verified to be larger than 6 dB while the OAM-AE is more than 40% for the generated seven OAM modes with various resonant heights. The experimental demonstration of the simulated results is carried out by fabricating four prototypes that generate OAM modes *l* = 0, −1, 2, −3, and the corresponding Wilkinson power divider (W-PD) is designed rationally using a novel design process to realize extended operating bandwidths for the fabricated prototypes. What’s more, the proposed arrangement design of the W-PD can be applicable for feasible OAM modes. The experimental results confirms that a gain enhancement of at least 6 dB is achieved within the 5% 3-dB gain bandwidth in the fabricated prototypes when generating four OAM modes. It should be noted that if the height location of the EBG is fixed appropriately for different OAM modes, a gain enhancement could also be obtained, although this is not optimal.

The proposed high gain OAM generation method not only offers the significant advantage of a reasonable and convincing high gain OAM prediction and a 3-dB gain bandwidth with concentration of different OAM mode’s main beam lobes, but also provides economic advantages including structural simplicity and ease of fabrication based on use of unprinted dielectric slabs with lower permittivity. Additionally, we can evaluate the radiation efficiency of each OAM mode utilizing the proposed analytical solution. The proposed design process is characterized by the fact that it achieves high gain OAM performance using a miniaturized array aperture and an easily-adjustable feeding system while also preserving the required OAM rotating phase front, which will become highly competitive in the future long distance OAM radio wave communication applications. Furthermore, since a homogeneous dielectric superstrate is applicable for dual-linearly or circularly polarization due to the structure symmetry, and therefore the proposed design, which is based on unprinted homogeneous dielectric slabs, has potential applications for an extension of a linearly polarized CAA to dual-linearly polarized and circularly polarized CAA with corresponding antenna element serves as the CAA element.

## Results

### Analysis of the EBG structure with oblique incidence

**Figure 1:**
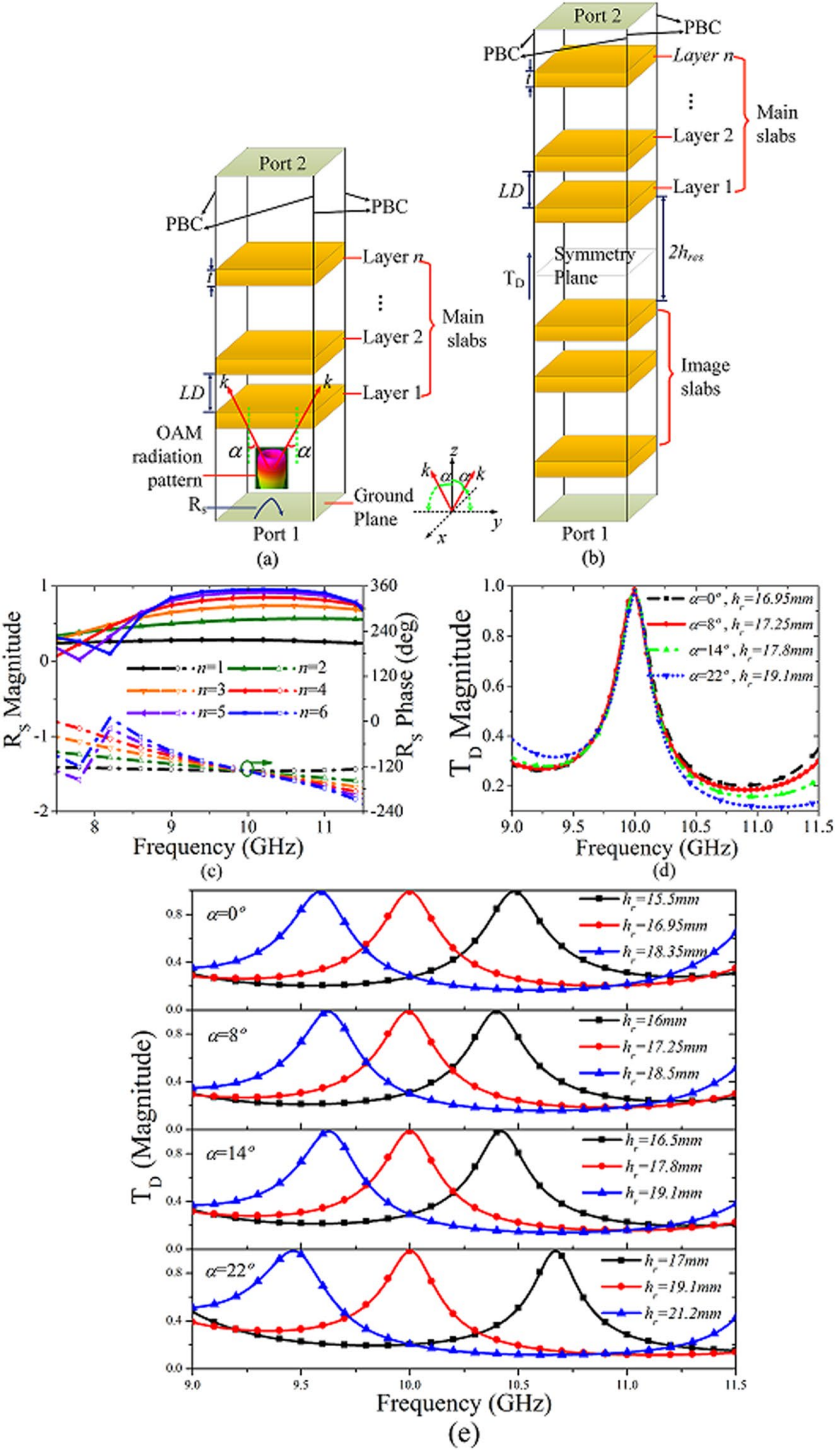
Simulation-based prediction of the high gain OAM performance with various main lobe directions based on the superstructure model (SM) and the defect cavity model (DCM) of the EBG structure when excited by an oblique incident plane wave with an incident angle *α*. (**a**) The schematics of the SM of the EBG structure consisting of *n* layers of unprinted slabs. (**b**) The schematics of the DCM of the EBG structure consisting of *n* layers of unprinted slabs and the corresponding image slabs. (**c**) Simulation of the effects of the number of layers *n* on the reflection phase and the reflection magnitude of the SM. (**d**) Simulated transmission magnitude of the DCM for *n* = 4 with respect to various incident angle *α* and varying resonant height *h*_*res*_ to achieve the optimal transmission magnitude T_D_ at the designed operating frequency of 10 GHz. (**e**) The detailed parametric analysis of the effects of the resonant height *h*_*res*_ on the transmission magnitude T_D_ of the DCM for *n* = 4 with incident angle *α* varying.

The OAM vortex wave is characterized by having an amplitude null region at the centre of the doughnut-shaped intensity profiles, and the divergent angle *θ* of the main beam increases with an increase in the OAM mode |*l*|, which will result in rapid wave divergence during long distance propagation. Based on the defect mode transmission theory, theoretical guidelines are proposed here for design of the EBG superstructure with oblique incidence to achieve vortex wave concentration and maximum gain enhancement. We begin by characterizing the EBG superstructure utilizing both the superstructure model (SM) and defect cavity model (DCM)^30,31^ excited by an obliquely incident plane wave with an incident angle *α* to achieve reasonable prediction of the high gain OAM performance. Schematics of the simulation models are shown in Fig. 1(a) and (b), with the periodic boundary conditions (PBCs) and Floquet ports utilized for the boundary setup in the commercial software High Frequency Structure Simulator with version of 2015 (ANSYS HFSS V.15). It should be noted that the DCM model is obtained by removing the ground plane in SM model and adding the image dielectric slabs of the main slabs. The initial superstructure of the EBG structure consists of *n* layers of unprinted slabs that were designed with low relative permittivity *ϵ*_*r*_ = 2.65, thickness *t* = 2 *mm*, and inter-slab separation *LD*.

Firstly, the SM is used to analyse the reflection coefficient R_S_ to ensure a high R_S_ magnitude of more than 0.6 for high gain achievement at the predetermined operating frequency of 10 GHz. The inter-slab separation *LD* is optimized as 11.5 mm (approximately *λ*_0_/2 at 10 GHz), and the number of the unprinted dielectric slab layers *n* is varied with an intersection of the R_S_ phase curves (with the R_S_ phase value of −129.1°) being obtained at the operating frequency of 10 GHz, as can be observed from Fig. 1(c). Figure 1(c) clearly shows that the *R*_*S*_ magnitude increases with increasing *n* up to *n* = 4, which means that *n* = 4 is an appropriate number of layers to achieve a sufficiently large *R*_*S*_ magnitude, which demonstrate the operating frequency of 10 GHz. It can be clearly observed from Fig. 1(c) that the R_S_ magnitude increases with *n* increase until *n* = 4, which means that *n* = 4 is appropriate for a sufficiently large R_S_ magnitude, and thus the construction of the EBG structure is determined. Based on the reciprocal space analysis^32^, the radially evanescent modes can be restricted to the broadside direction by introduction of a defect band with an EBG structure. Therefore, once the construction of the EBG structure is determined, the DCM is then utilized to analyze the defect mode bandwidth (DMB, T_D_ > 0.7) around 10 GHz for the prediction of the EP-CAA’s 3-dB gain bandwidth^31^. The results of a detailed parametric analysis of the effect of the resonant height *h*_*res*_ on the transmission magnitude T_D_ with varying incident angle *α* varying for *n* = 4 are shown in Fig. 1(e).

The DMB is observed to shift to lower frequency as the resonant height *h*_*res*_ increases for different incident angles *α*, and the corresponding resonant height *h*_*res*_ increases with increasing *α* to achieve a consistent 3-dB gain bandwidth around 10 GHz. As has been concluded in Fig. 1(d), the optimized values of resonant height *h*_*res*_ that correspond to incident angles *α* = 0°, 8°, 14°, 22°, are 16.95 *mm*, 17.25 *mm*, 17.8 *mm*, 19.1 *mm*, respectively, for a consistent 3-dB directivity bandwidth around 10 GHz (9.87–10.11 GHz, DMB of 2.4%), which means that an increasing resonant height *h*_*res*_ is essential for a consistent 3-dB directivity bandwidth around 10 GHz with the oblique incidence increasing. In addition, it should be noted that with an appropriate constant resonant height *h*_*res*_, the gain enhancement at different incident angle can also be obtained with a suitably high R_S_ magnitude of >0.6, but it is not optimal, unlike the value obtained using the varied resonant height *h*_*res*_ with incident angle *α* as shown in Fig. 1(d).

### Detailed analysis of high gain OAM beams generated based on the proposed EP-CAA

**Figure 2:**
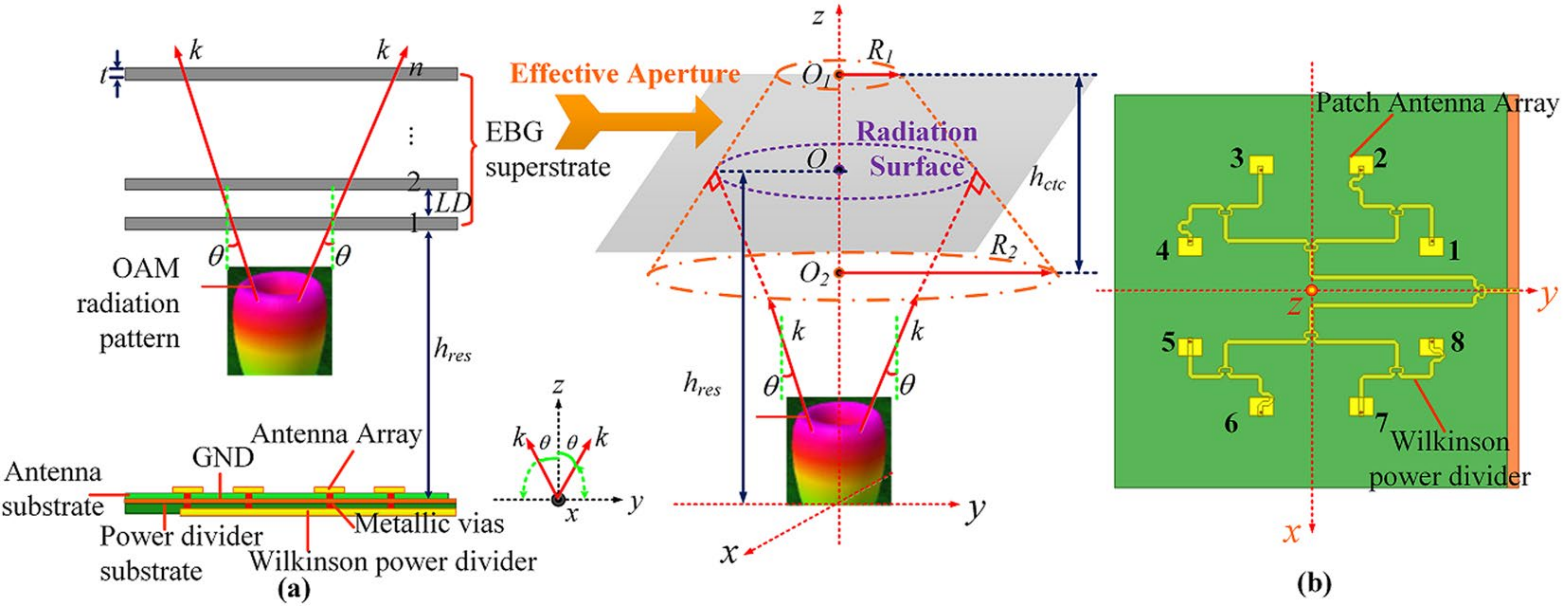
(**a**) Lateral view and definition of the effective radiation aperture of the proposed high gain OAM generator EP-CAA. (**b**) The front perspective view of the original CAA when operating at OAM mode *l* = 2.

As presented in the previous section, the EBG superstrate is well-designed, and the proposed EP-CAA is then configured by the integration of a microstrip patch CAA and the well-designed parasitic EBG superstrate, as illustrated in Fig. 2(a). The CAA is adopted here to generate OAM vortex waves along the propagation direction of *z*-axis. The utilization of the EBG superstrate loaded upon the CAA is intended to modulate the propagating *E*-field distribution for a much more uniform presentation and thus achieve the required substantial gain enhancement. The CAA consists of two stacked substrate layers with relative permittivity *ϵ*_*r*_ = 2.65 and loss tangent 0.0013. The antenna array is printed on the top surface of the upper antenna substrate with a thickness of 0.8 *mm*, while the W-PD is printed on the bottom surface of the lower power divider substrate with a thickness of 1 *mm*. Since the W-PD is characterized by its simple structure, ease of adjustment and wideband operation^33^, it is then adopted here to act as the feed network to provide the desired successive phase shift of 2*πl*/*N* (where *N* is the number of antenna elements) for various OAM modes while employing metallic vias to feed the antenna array. Furthermore, the distinctive wideband operation of the W-PD can also be used to further extend the 3-dB directivity bandwidth of the proposed EP-CAA. The ground plane (GND) is inset in the middle of the two stacked substrate layers to provide a common ground for both the antenna array and the power divider. Dimensions of the GND designed with 180 × 185 *mm* are slightly larger than dimensions of the antenna substrate, which are set as 180 × 185 *mm* for stable loading of an SMA connector. Dimensions of the EBG-superstrate are consistent with the GND, and the aperture radius of the EBG is then defined as R_*EBG*_ = 185 mm/2. It should be noted here that the designed two-layer CAA structure with the GND layer positioned in the middle has advantages of greatly reduced dimensions and the elimination of spurious radiation^34,35^. The front perspective view of the original CAA operating at OAM mode *l* = 2 is presented in Fig. 2(b) to provide a better description of the original CAA prototype. As shown in Fig. 2(a), the distinctive divergent OAM wave produced by the CAA is incident on the EBG superstrate with a divergent angle *α*. It should be emphasized here that the divergent angle of the OAM wave is symmetrical relative to the *z* axis, which will result in a reduced effective aperture area with an increased field amplitude null region in the radiation centre when the OAM mode number is increasing. Therefore, the corresponding OAM-AE will decrease as the OAM mode increases. Since the antenna radiation aperture is defined as the aperture that is perpendicular to the main lobe of the incident electromagnetic wave, the corresponding effective aperture area of the effective radiating EBG area is then defined as the lateral area of a circular truncated cone with an upper and bottom surfaces of radius *R*_1_ = *h*_*res*_ · tan^2^ *θ* and *R*_2_ = *R*_1_ + (R_*EBG*_ − *h*_*res*_ · tan*θ*) · cos^2^ *θ*, respectively. And the corresponding height of the lateral area is *h*_*ctc*_ = *R*_1_ · cot*θ* + (R_*EBG*_ − *h*_*res*_ · tan*θ*)·cos*θ*·sin*θ*. Then, the area of the circular truncated cone ${A}_{effective}=\pi \cdot \sqrt{{({R}_{2}-{R}_{1})}^{2}+{{h}_{ctc}}^{2}}\cdot ({R}_{1}+{R}_{2})$ can be deduced for an exact and analytical calculation of the OAM-AE. The corresponding effective aperture is plotted on the right side of Fig. 2(a) for a concise explanation, it should be noted that the centres of the upper and bottom surfaces of the circular truncated cone, which are defined as O_1_ and O_2_, respectively, are located on the *z*-axis. For a better illustration, the radiation surface on the EBG aperture that is formed by the main lobes of an OAM beam with the center of O is also located on the *z*-axis. Eight patch antenna elements are arranged in a circular array to constitute the original CAA, and the radius of the CAA is optimized as 60 mm (about 2*λ*_0_) to generate relatively directive OAM radiation patterns for a higher OAM-AE and relatively low inter-element mutual coupling. The dimensions of the patch antenna element are set at 13 × 8 *mm* for the operation around 10 GHz, and the EM wave polarization is along *x*-axis.

**Figure 3:**
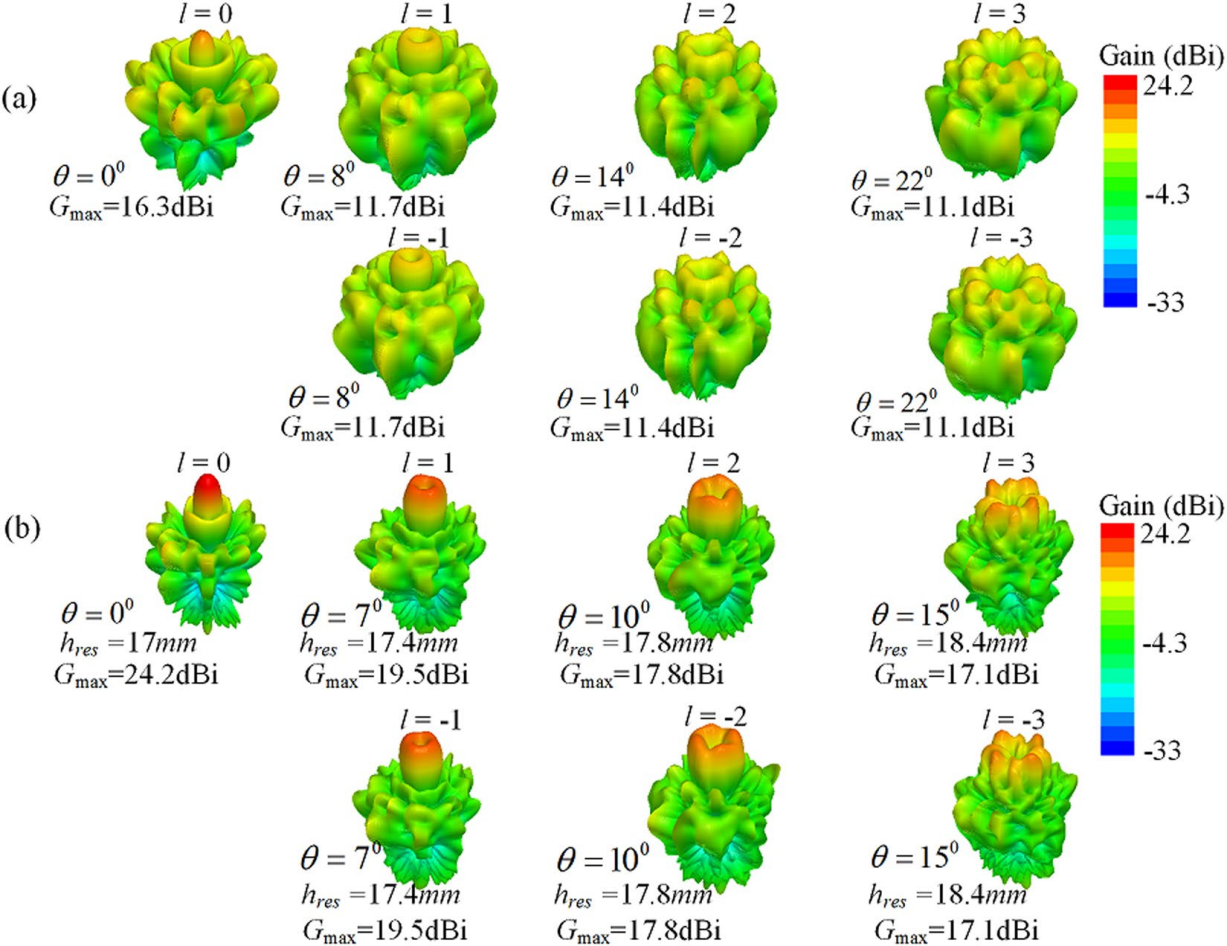
Comparison of simulated far-field radiation patterns of the original CAA and the proposed EP-CAA without the W-PD for different OAM modes. (**a**) The original CAA, and (**b**) the proposed EP-CAA operating at 10 GHz. It should be noted here that the resonant height *h*_*res*_ of the EP-CAA differs for various OAM modes.

The overall structure is firstly analysed without the PD for simplicity and feasibility, the design procedure for the W-PD will be presented in detail later for the experimental verification of the theoretical analysis. The far-field radiation characteristics of the original CAA carrying seven OAM modes are simulated to obtain the main beam directions *θ*, which are observed as 22°, 14°, 8°,0°, 8°, 14°, 22° with maximum realized gain *G*_max_ values of 11.1, 11.4, 11.7, 16.3, 11.7, 11.4, 11.1 dBi for OAM modes *l = *−3, −2, −1, 0, 1, 2, 3, respectively. The OAM modes with the same |*l|* are observed to have the same main beam direction *θ* and the identical maximum realized gain *G*_max_, and thus only positive OAM modes are considered for simplicity in the following sections. Based on the discussions in the previous section, the resonant distance *h*_*res*_ increases with increasing main beam direction angle *θ*, which means that *h*_*res*_ should be different for various OAM modes |*l|*. According to the previous analysis of the EBG structure, values of resonant distance *h*_*res*_ are initially set as 16.95, 17.25, 17.8, 19.1 *mm* for OAM modes |*l| = *0, 1, 2, 3, respectively, and *θ* are observed as 0°, 8°, 14°, 22°, respectively. Since the construction of the EP-CAA differs from the simulated EBG structure due to the limited area of the EBG structure and the different radiation elements, values of resonant distance *h*_*res*_ are then optimized as 17, 17.4, 17.8, and 18.4 *mm*, for OAM mode |*l| = *0, 1, 2, 3, respectively, to achieve the maximum gain enhancement for the EP-CAA. As shown in Fig. 3(b), the maximum gain *G*_max_ for the EP-CAA has values of 24.2, 19.5, 17.8, 17.1dBi with *θ* concentrated at 0°, 7°, 10°, 15° for |*l| = *0, 1, 2, 3, respectively, which corresponding to gain enhancements of 7.9, 7.8, 6.4, and 6.0 dB, respectively. The far-field divergence angles of different OAM modes are effectively improved when compared with the original CAA, and the corresponding OAM-AE is increased from 33.7%, 20.1%, 20%, and 20.4% to as high as 83.7%, 49.4%, 41.8%, and 40.9% for |*l| = *0, 1, 2, 3, respectively. It should be noted that the present high gain OAM generator is achieved using a miniaturized array aperture.

**Figure 4:**
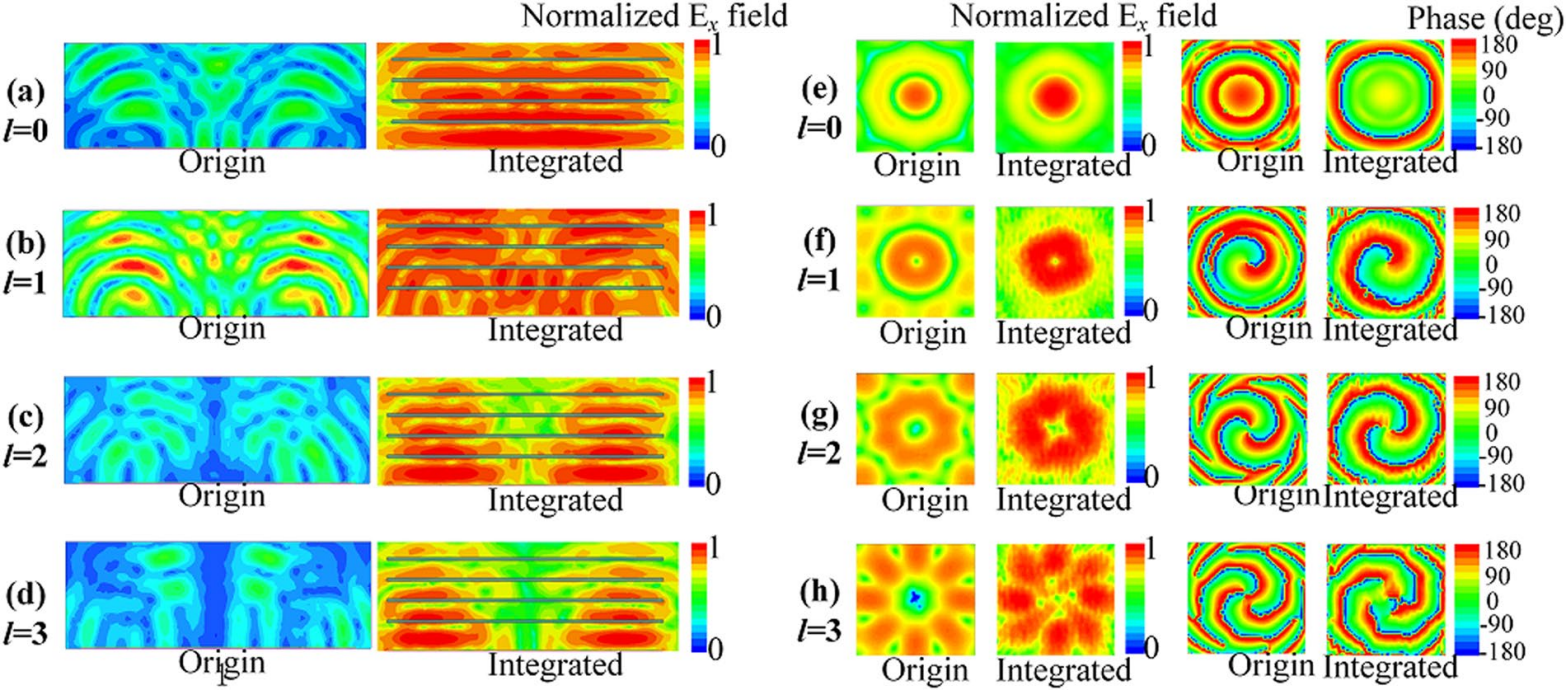
Comparison of the normalized electric field distribution on *xoz* plane of the original CAA and the EP-CAA without the W-PD generating OAM mode, (**a**) *l* = 0, (**b**) *l* = 1, (**c**) *l* = 2, (**d**) *l* = 3, and the comparison of the near-field magnitude and phase distributions of the electrical field *E*_x_ on the near-field sampling plane of the original CAA and the EP-CAA for different OAM modes, (**e**) *l* = 0, (**f**) *l* = 1, (**g**) *l* = 2, (**h**) *l* = 3.

For a better demonstration of the working principle of the proposed high gain OAM vortex wave generator, a comparison of the electric field distributions on *xoz* plane and on the near-field sampling plane of both the original CAA and the EP-CAA for OAM modes *l* = 0, 1, 2, 3 are shown in Fig. 4. The near-field sampling plane is located above the GND at the distance of 500 *mm*. The electric field magnitude distributions plotted on *xoz* plane and the near field scanning plane are normalized with respect to the maximum electric field magnitude of the corresponding EP-CAA. The figures labelled “Origin” indicates the field distributions of the original CAA, while the figures labelled “Integrated” indicates the field distribution of the EP-CAA. In contrast with the original CAA, a much more uniform field distribution and almost in phase outgoing wave front on *xoz* plane are observed for the EP-CAA when generating OAM mode 0, and a larger electric field magnitude along with an obvious wave front shaping can also be shown in Fig. 4 (a)–(d). And thus, the observed phenomenon is responsible for achievement of the high-gain OAM and the higher OAM-AE.

**Figure 5:**
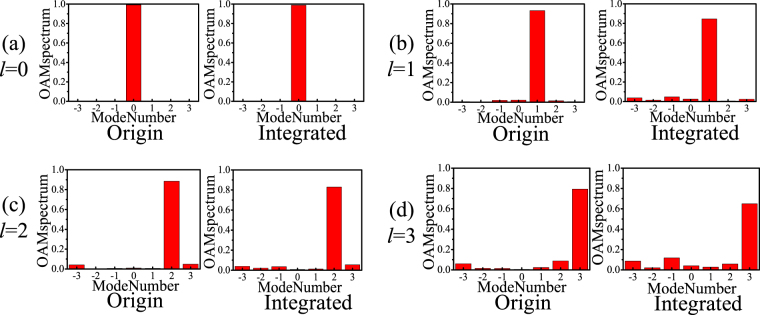
The corresponding power distributions at different OAM modes. (**a**) *l* = 0, (**b**) *l* = 1, (**c**) *l* = 2, (**d**) *l* = 3.

Furthermore, the magnitude null regions at the centres of the near-field magnitude distributions are observed to increase with increasing OAM mode *l* for the original CAA, which demonstrates the deterioration in OAM transmission for long distance propagation. With the integration of the EBG superstrate at the optimized resonant height *h*_*res*_, we can see that the near field magnitude distribution of the EP-CAA shows a significant field concentration around the radiating beam centre for all OAM modes when compared with the original CAA. As a result, the maximum *E*-field magnitude of the EP-CAA is enhanced remarkably, and the near field distribution is more uniform when compared with that of the original antenna array. In addition, the comparison of the phase distributions of the original CAA and the integrated EP-CAA shows that stable OAM vortex phase fronts are maintained. The corresponding power distributions at different OAM orders is analysed based on the purity calculation of OAM modes^36^. As shown in Fig. 5, the mode purity for the original CAA generating OAM modes *l* = 0, 1, 2, 3 are 0.99, 0.93, 0.89, 0.79, respectively, while the mode purity for the EP-CAA generating OAM modes *l* = 0, 1, 2, 3, are 0.99, 0.85, 0.83, 0.7, respectively. It can be observed that the purities of the EP-CAA have been decreased by 0.3%, 8.6%, 6.7%, 11.4% for OAM modes *l* = 0, 1, 2, 3, respectively, when compared with the original CAA. In general, the mode purity of the generated OAM waves using EP-CAA still remains good for the already generated OAM states. On the other hand, the maximum mode crosstalk for the original CAA generating OAM modes *l* = 0, 1, 2, 3, are 0.002, 0.02, 0.05, 0.09, respectively, while the maximum mode crosstalk for the EP-CAA generating OAM modes *l* = 0, 1, 2, 3 are 0.003, 0.05, 0.05, 0.09, respectively. In general, the mode purity of both the original and integrated cases remain larger than 0.7, while the mode crosstalk remains lower than 0.1, which demonstrate that our proposed method can achieve a high gain performance with the OAM mode purity substantially retained. Therefore, the proposed high gain, high aperture efficiency OAM vortex wave generation method is demonstrated through simulations to improve the transmission efficiency significantly for long distance transmission.

### Experimental results and the detailed design of the proposed W-PD arrangement

**Figure 6:**
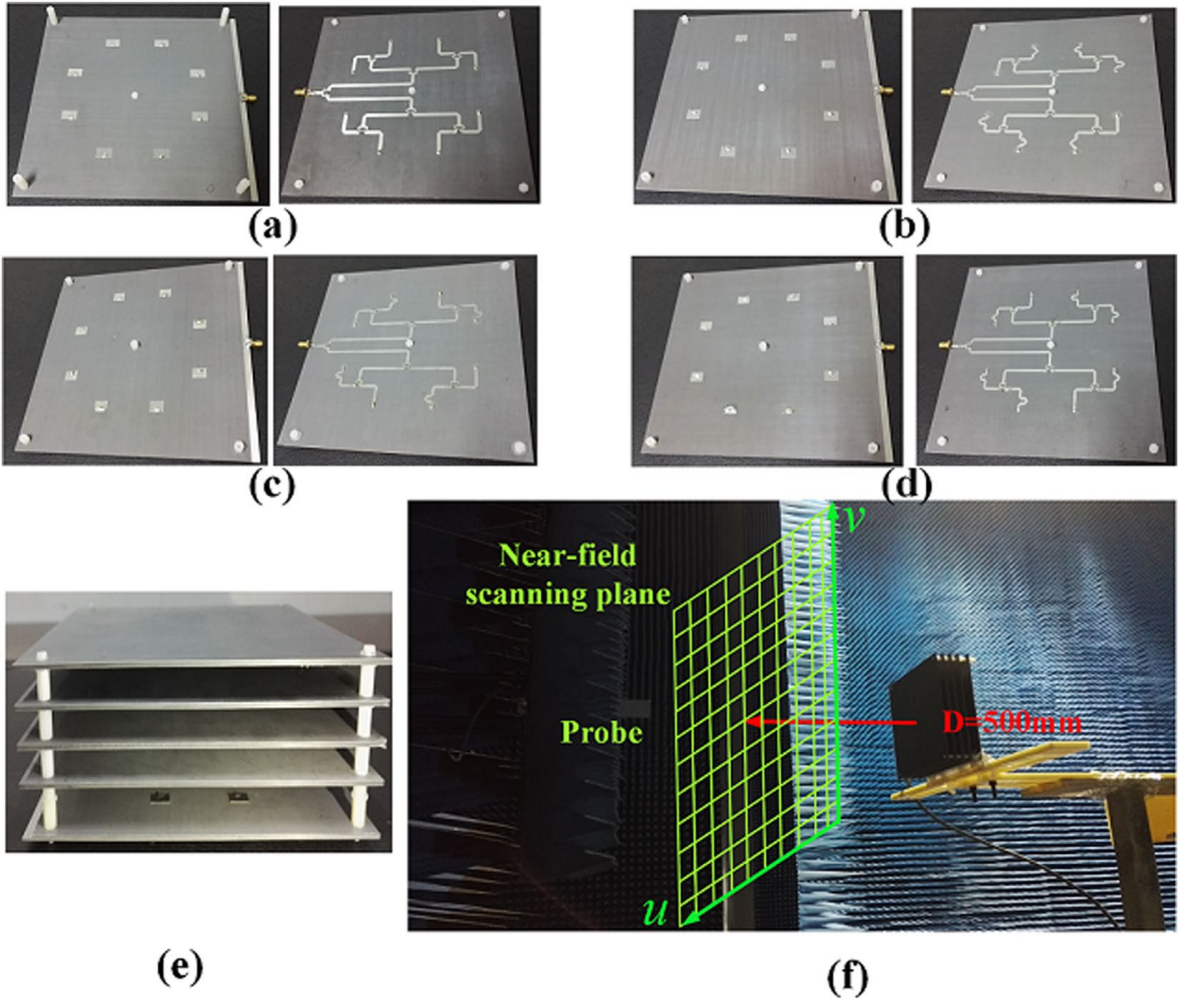
Fabricated prototypes of the proposed EP-CAA. Front view and back view of the original CAA when generating OAM modes (**a**) *l* = 0, (**b**) *l* = −1, (**c**) *l* = 2, (**d**) *l* = −3. (**e**) Solid view of the EP-CAA, and (**f**) experimental system configuration for the OAM vortex wave measurement using near-field scanning technique.

**Figure 7:**
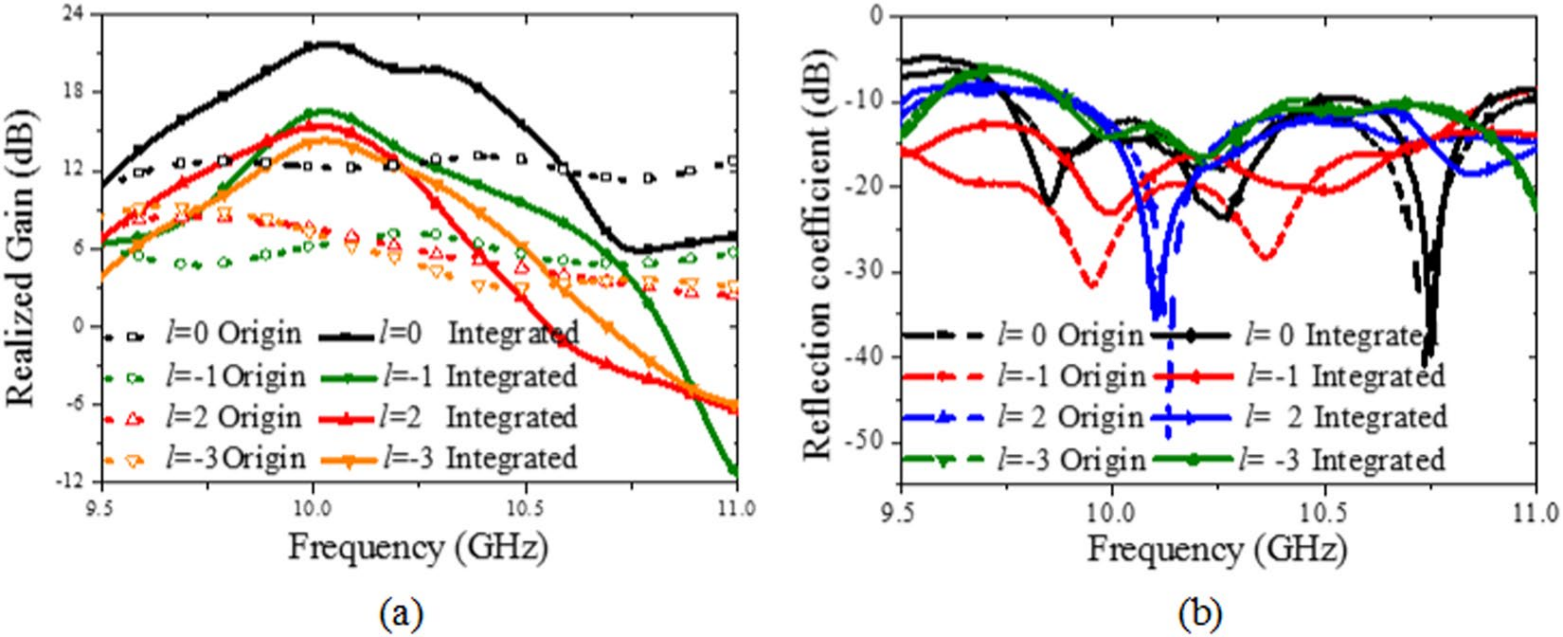
(**a**) Simulated 3-dB gain bandwidths and (**b**) measured reflection coefficients of the fabricated prototypes with the well-designed W-PD when generating OAM modes *l* = 0, −1, 2, −3.

To verify the effectiveness of the high gain OAM design method experimentally, prototypes of the EP-CAA generating OAM modes *l* = 0, −1, 2, −3 are fabricated and measured, as shown in Fig. 6. In this paper, the arrangements of the CAA and the W-PD are designed in a concise manner to form the OAM modes |*l*| = 0, 1, 2, 3 accordingly. It is remarkable that the proposed design method for the antenna arrangement is feasible to generate arbitrary OAM mode. After careful deliberation and repeatedly verification, it can be concluded that the CAA arrangement should be either symmetrical or anti-symmetrical about the centre axes (*x* axis and *y* axis), which would allow the far-field radiation pattern obtained to be symmetrical. To illustrate the proposed arrangement in detail, an 180° inversion of antenna elements 5, 6, 7, 8 for generation of the OAM mode *l* = −1, an inversion of antenna elements 1, 4, 5, 8 for the mode *l* = 2, and an inversion of antenna elements 2, 3, 5, 8 for the mode *l* = −3 are carried out to reduce the feed blockage effect and shorten the phase-shift lines of the W-PD. Since the rotational phase fronts of the positive OAM modes *l* are reversed when compared with those of the negative OAM modes, feeding networks for the negative OAM modes can be easily obtained by taking the mirror image from the feeding networks of the positive OAM modes while maintaining the same arrangement of antenna elements. A front perspective view of the CAA for generation of the OAM mode *l* = 2 is shown in Fig. 2(b) for simplicity, and the overall constructions of the CAAs for generation of OAM modes *l* = 0, −1, 2, −3 are presented as fabricated prototypes as shown in Fig. 6. The simulated phase shifts between adjacent ports are highly consistent with the theoretically calculated ones with the maximum phase variation of 3°, and the amplitudes of all the ports are almost equal to each other with the maximum amplitude variation of 0.5 *dB*. The consistent power delivery helps to guarantee the purity of OAM modes and provides reduced gain losses for the EP-CAA. The experimental system configuration used for the near-field measurements is shown in Fig. 6(c). The near-field scanning plane, with its dimensions of 500 × 500 *mm* and sampling grid period of 10 *mm*, is placed at *D* = 500 *mm* far away from the antenna array. The horizontal polarization component of the radiated electric field *E*_*u*_ is detected using a standard measuring probe on the sampling plane during the measurements. Reflection coefficients of the original CAAs and of the EP-CAA are measured to demonstrate good operation in the frequency range around 10 GHz, with an extended bandwidth of 9.8% (9.7~10.7 GHz) produced by adoption of the W-PD, as shown in Fig. 7(b). As presented in Fig. 7(a), the corresponding 3-dB gain bandwidth is verified through simulation to be as wide as 5% for every OAM mode that is fabricated, which is apparently wider than the predicted one utilizing the EBG structure. The application of the W-PD has thus been demonstrated to enhance the bandwidth performance in terms of both the return loss and 3-dB gain through both simulations and experiments.

**Figure 8:**
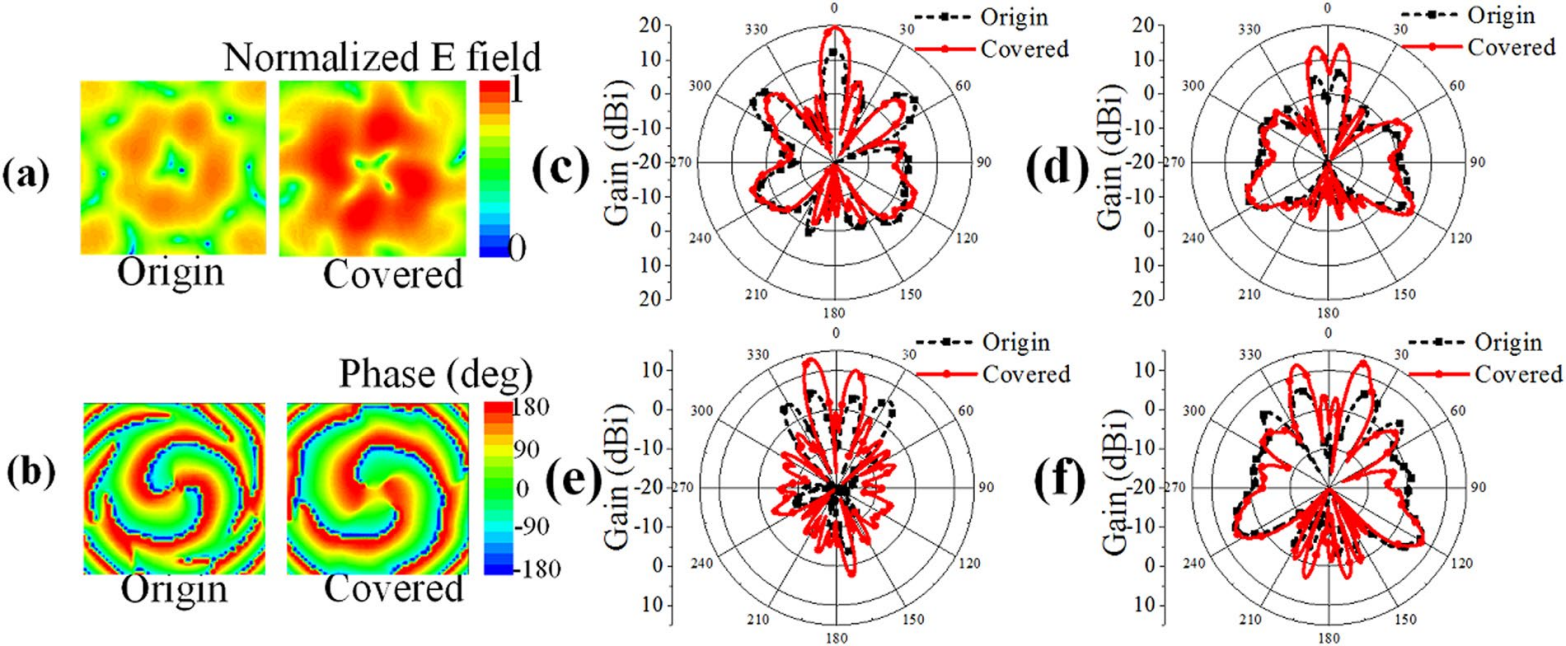
Comparison of the measured near-field distributions and far-field radiation patterns of the original CAA and the EP-CAA. (**a**) Near-field magnitude distribution of OAM mode *l* = 2. (**b**) Near-field phase distribution of OAM mode *l* = 2. (**c**) Far-field radiation pattern of OAM mode *l* = 0. (**d**) Far-field radiation pattern of OAM mode *l* = −1. (**e**) Far-field radiation pattern of OAM mode *l* = 2. (**f**) Far-field radiation pattern of OAM mode *l* = −3.

**Figure 9:**
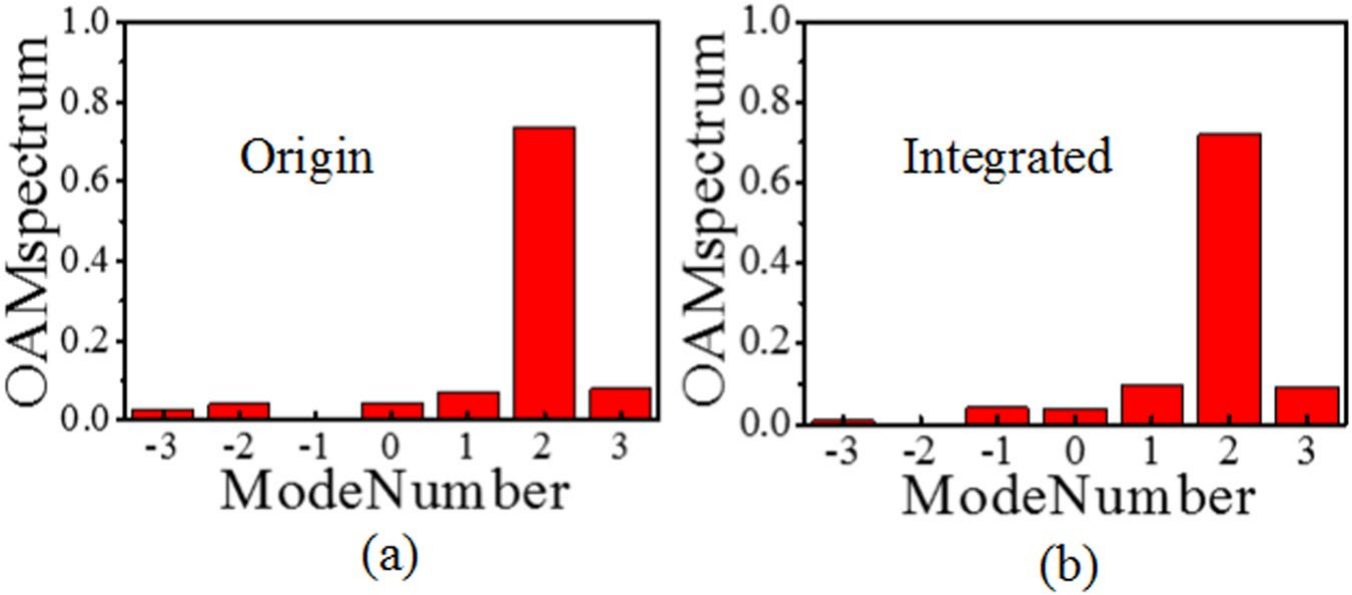
The corresponding power distributions at the measured OAM order *l* = 2.

The measured 2D far-field radiation patterns and the near-field magnitude and phase distributions of *E*_*u*_ for the OAM mode *l = *2 are shown in Fig. 8 at the operating frequency of 10 GHz. A doughnut-shaped intensity map and a rotating phase front can clearly be observed in the measured near-field distribution, and these are typical characteristics of OAM waves. As shown in Fig. 8, both the divergent OAM near-field magnitude distribution and the far-field radiation pattern of the EP-CAA can be effectively concentrated with an enhanced gain performance when compared with that of the original CAA. It can also be observed that the measured gain enhancement values are 8.0, 7.9, 7.5, 6.2 dB for *l = *0, −1, 2, −3, respectively, and the rotating phase front is maintained when compared with that of the original CAA. A gain improvement of at least 6 dB and remarkably concentrated main beam angles for the covered EP-CAA can be observed in the experimental results, which verify the theoretical analysis and the design for high-gain OAM vortex wave generation. As shown in Fig. 9, in experiment, the maximum mode purity for the origin and integrated cases generating OAM mode 2 are 0.74, 0.72, respectively, and the maximum mode crosstalk for the origin and integrated cases are 0.08, 0.09, respectively. The phenomenon that the mode purity is slightly lowered than the simulated ones is due to the noise environment of the experiment. In general, both the theoretical and experimental results prove that the mode purity of the original and integrated cases remain larger than 0.7, and the mode crosstalk remains lower than 0.1, which demonstrate that our proposed method can achieve a high gain performance with the OAM mode crosstalk substantially retained at an enough low level. The measured results are slightly different from the simulated ones, which is partly due to the fact that the simulated EP-CAA is designed without the W-PD while the experimental devices are fabricated with four different W-PDs. In addition, the fact that the practical loading resonant height *h*_*res*_ in the experiments cannot have the exact value of the height in the simulation, and the fabrication errors caused by the welding of the CAA and the W-PD, also result in undesired measurement differences with the simulated results.

## Discussion

In this paper, we present an effective high gain OAM vortex wave generation approach based on the integration of a CAA with an EBG superstrate. Simulations and measurements show that the gain enhancement of the CAA when integrated with the EBG superstrate is at least 6 dB for all the OAM modes when compared with the original CAA. Theoretical principles are deduced for the design of the EBG superstrate and determination of the resonant height for maximum gain enhancement with a reliable prediction of 3-dB gain enhancement for different oblique angles of incidence. It should be noted that if the location of the EBG superstrate is properly fixed for different OAM modes, the gain enhancement could also be obtained, although it may not be optimal. The effective radiation aperture of the EP-CAA with an oblique angle of incidence is proposed and defined for analytical calculation of the aperture efficiency of the OAM beams (OAM-AE), which acts as a very useful guideline for evaluation of the effective OAM radiation efficiency. The proposed method is beneficial for long-distance transmission of OAM vortex waves. Furthermore, as demand continues to grow for higher transmission efficiency in communications, the wide band and hybrid mode OAM will inevitably become a developing research trend, and thus the achievement of high gain OAM performance in wide-band and for hybrid OAM mode will be very attractive for utilization in long distance and high-efficiency transmission applications. Based on the proposed method of applying the defect mode transmission to achieve high gain OAM performance, the method can also be extended to applications in wider bands, the hybrid OAM mode for a valuable in-depth research.

## Methods

The numerical simulation of the EBG structure during stimulation by obliquely incident waves is based on the driven mode, in which the Floquet ports and master/slave boundary conditions are used. The electric field distribution is obtained using the HFSS-IE domain boundary for a reduction of the simulation calculation. The EP-CAA is manufactured using the printed circuit board (PCB) technology. The measurements are carried out using a 3D platform in an anechoic chamber based on the Agilent Vector Network Analyzer E8363B. The return loss, the near-field distribution and the far-field radiation patterns of the proposed EP-CAA are measured.
